# Gene Therapy of the Hemoglobinopathies

**DOI:** 10.1097/HS9.0000000000000479

**Published:** 2020-09-11

**Authors:** Joachim B. Kunz, Andreas E. Kulozik

**Affiliations:** Department of Pediatric Oncology, Hematology and Immunology, University of Heidelberg, Hopp – Children's Cancer Center Heidelberg (KiTZ), Heidelberg, Germany.

## Abstract

Sickle cell disease and the ß-thalassemias are caused by mutations of the ß-globin gene and represent the most frequent single gene disorders worldwide. Even in European countries with a previous low frequency of these conditions the prevalence has substantially increased following large scale migration from Africa and the Middle East to Europe. The hemoglobin diseases severely limit both, life expectancy and quality of life and require either life-long supportive therapy if cure cannot be achieved by allogeneic stem cell transplantation. Strategies for *ex vivo* gene therapy aiming at either re-establishing normal ß-globin chain synthesis or at re-activating fetal γ-globin chain and HbF expression are currently in clinical development. The European Medicine Agency (EMA) conditionally licensed gene addition therapy based on lentiviral transduction of hematopoietic stem cells in 2019 for a selected group of patients with transfusion dependent non-ß° thalassemia major without a suitable stem cell donor. Gene therapy thus offers a relevant chance to this group of patients for whom cure has previously not been on the horizon. In this review, we discuss the potential and the challenges of gene addition and gene editing strategies for the hemoglobin diseases.

## The clinical challenges of hemoglobinopathies

Hemoglobinopathies, most importantly sickle cell disease (SCD) and ß-thalassemia, are the most prevalent genetic disorders globally.^1,2^ Migration from regions of high prevalence has resulted and will likely continue to result in a rise of patient numbers in Europe.^3,4^

Thalassemia mutations either completely abolish (ß^0^) or substantially reduce (ß^+^) the expression of the hemoglobin ß-chain, resulting in an imbalance of the hemoglobin α- and ß-chains. This imbalance causes hyperplastic but ineffective erythropoiesis and can result in extramedullary hematopoiesis.^5^ While the ß-thalassemias are genetically diverse with various mutations being prevalent among different ethnic groups, the clinical phenotype in most patients with biallelic inactivation of the ß-globin gene is that of thalassemia major, characterized by the need for life-long regular red blood cell transfusions from early childhood (TDT, “transfusion dependent thalassemia”). Approximately 10% to 15% of patients who carry either mutations allowing for relevant residual expression of ß-globin, who carry genetic modifiers boosting the expression of γ-globin and fetal hemoglobin (HbF) beyond infancy or who have co-inherited α-thalassemia mutations present with the phenotype of thalassemia intermedia. These patients may require red blood cell transfusions only occasionally, but typically develop skeletal changes and iron overload later in life because of marked erythroid hyperplasia. Recently, this clinical phenotype has also been referred to, probably inappropriately, as “non-transfusion dependent thalassemia” (NTDT).^6^

The mainstay of thalassemia treatment is red blood cell transfusion, accompanied by intensive iron chelation. While most patients will reach adulthood if well treated, both, quality of life and life expectancy are severely impaired by the complications of iron overload.^7–9^ If an HLA-matched stem cell donor is available, allogeneic stem cell transplantation as soon as possible and before iron overload develops is considered advisable.^10–12^ The economic burden of thalassemia is substantial especially in countries of high prevalence. The expenses for red blood cell transfusions and for iron chelation are estimated to exceed €30,000 annually for an adult patient.^8,13^

Patients with SCD always carry the HbS allele that codes for a valine instead of a glutamic acid residue at position 6 of ß-globin, either in a homozygous or a compound heterozygous state, and consequently express exclusively or predominantly sickle cell hemoglobin, HbS. Valine, a hydrophobic amino acid, at position 6 favors the polymerization of deoxygenated HbS and the formation of sickle-shaped erythrocytes. After the hemoglobin switch, when HbF is replaced by HbS during infancy, patients present with chronic hemolysis, acute vasoocclusive crises and a chronic vasculopathy that affects all organs.^14^ As is the case for ß-thalassemia, the severity of SCD is modulated by the expression of HbF and the co-inheritance of α-thalassemia. This modulation is most obvious in patients who carry, besides HbS, one allele of the ß-globin gene cluster that does not turn off the expression of HbF during infancy (“hereditary persistence of fetal hemoglobin”, HPFH).^15^ As HbF efficiently interferes with the polymerization of HbS, these patients can show few or no symptoms of SCD.^16^ Further, pharmacologic induction of HbF is known to reduce the severity of SCD.^17,18^ However, it must be noted that even high levels of HbF do not consistently improve all complications of SCD.^16,19^

Without adequate medical care, most patients with SCD die during early childhood.^20,21^ With the use of prophylactic antibiotics, vaccinations, parent education, assessment for risk of stroke by transcranial Doppler ultrasound, red blood cell transfusions and hydroxycarbamide, more than 95% of patients reach adulthood.^22,23^ Nevertheless, the quality of life is severely impaired by painful vasoocclusive crises and chronic organ damage. Life expectancy, even with optimal care, is shortened by approximately two decades.^24–28^ During recent years, pharmacologic treatments targeting several pathophysiologic mechanisms of SCD have been developed and show clinically relevant effects.^29–33^ Although combinations of these drugs can potentially offer a chance for a meaningful reduction of SCD symptoms in many patients, they do not offer cure and need to be applied over many years or even for the entire life. Currently, the only curative treatment is allogeneic stem cell transplantation which is increasingly considered as “standard of care” if an HLA-matched sibling donor is available.^11,34,35^ Stem cell transplantation from alternative donors, that is matched unrelated or mismatched family donors, is associated with a high morbidity and even a mortality in the range of 10% and is reserved for selected patients with a severe course of SCD.^36–38^

## Principles of gene therapy for the hemoglobinopathies

SCD and TDT are monogenic disorders and particularly amenable to gene therapy because the genetic defect needs to be corrected in hematopoietic stem cells giving rise to erythroid precursors only. With a history of allogeneic stem cell transplantation over more than 50 years,^39,40^ methods for the collection and manipulation of hematopoietic stem cells are readily available. Gene therapy^41^ offers the potential of cure for patients with TDT and with SCD even if no suitable stem cell donor is available and is not associated with the risk of Graft-versus-Host-Disease (GvHD). A tailored conditioning without the need for immunosuppression promises a reduced risk for short- and long-term toxicity in comparison to allogeneic stem cell transplantation.

**Figure 1:**
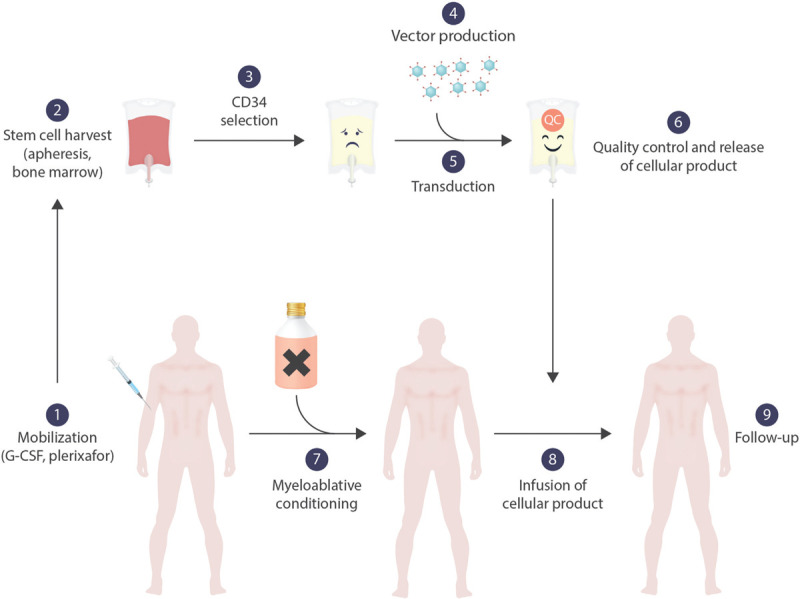
***Ex vivo* gene therapy for hemoglobinopathies.** Stem cells are mobilized by G-CSF and/or plerixafor (1) and harvested by leukapheresis (2). Alternatively, bone marrow without preceding mobilization can be harvested. Stem cells are enriched from the apheresis product by positive selection for CD34 (3). The vector is manufactured under GMP-conditions (4) and incubated with the purified stem cells (5). After the product has been subjected to rigorous quality controls and is released (6), the patient is treated with myeloablative chemotherapy (7). The manipulated stem cell product is applied either intravenously or intraosseously (8).

Gene therapies for the hemoglobinopathies in clinical use or clinical development all rely on *ex vivo* manipulation of hematopoietic stem cells and resemble autologous stem cell transplantation (Fig. 1).^42–44^ Several conditions must be met for the cure of a hemoglobinopathy. First, a sufficient number of hematopoietic stem cells must be obtained and manipulated without damaging or reducing their capacity of engraftment, self-renewal, proliferation and differentiation. Second, the gene defect must be corrected in a large majority of stem cells. Third, the manipulated stem cells have to be given into an environment that promotes survival and long term expansion, requiring myeloablative conditioning. A characteristic of thalassemias is the limited maturation potential of erythroid precursors, conferring a selective advantage to those precursors whose genetic defect has been repaired by gene therapy. Such an effect was first demonstrated after allogeneic stem cell transplantation for thalassemia, when a proportion of 20% of all nucleated bone marrow cells suffices to produce exclusively donor-derived erythrocytes.^45^ However, this selective advantage is by far less pronounced as compared to that of corrected T-cells in severe combined immunodeficiency- one of the reasons, why historically immunodeficiencies were the first targets of gene therapy.^46–48^

## History of gene therapy

**Figure 2:**
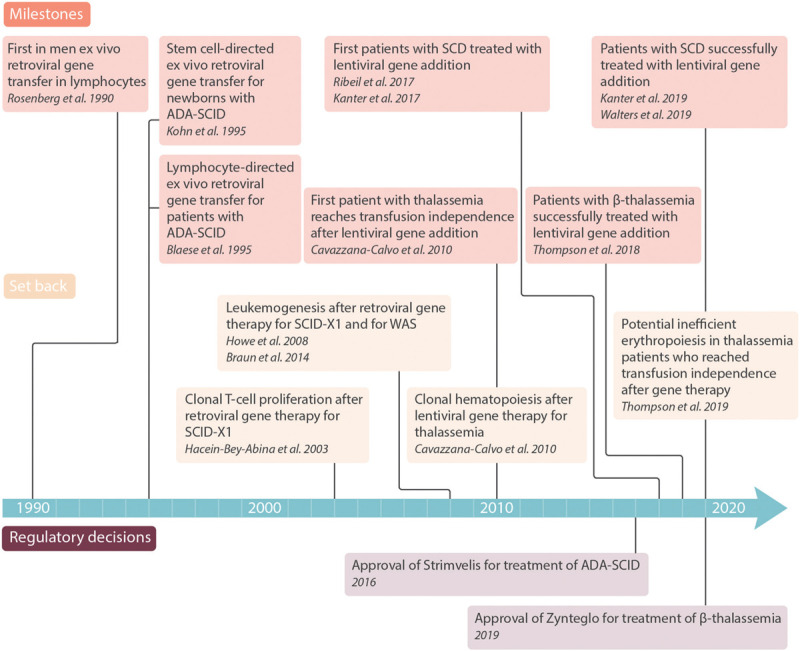
**Selected milestones of *ex vivo* gene therapy for hematologic disorders.**

With the advent of recombinant DNA-technologies and the cloning of ß-globin as first human gene in the mid-70ies^49^ very early and, from today's perspective, premature attempts for gene therapy of thalassemia were made.^50^ Because major obstacles—efficiency, longevity, and safety of gene transfer into hematopoietic stem cells—could not be overcome at that time, gene therapy for thalassemia and SCD remained a hypothetical option that had not become reality for decades (Fig. 2).

## γ-retroviral vectors

**Figure 3:**
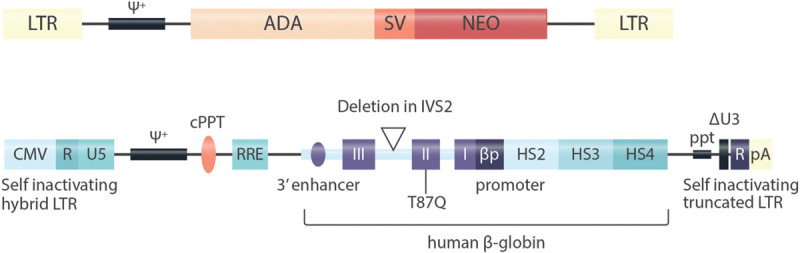
**Vectors used for gene addition.** A: The vector used for the first clinical application of gene transfer into human hematopoietic stem cells.^51^ This vector was based on the Moloney murine leukemia virus. LTR- long terminal repeat, ψ^+^ extended packaging signal; ADA, human ADA cDNA; SV, SV40 early region promoter; NEO, neomycin resistance gene. The viral LTR sequencing mediate high-level expression of the transgene and genomic integration in the vicinity of transcriptionally active genes, resulting in the risk of oncogene activation. B: The self-inactivating lentiviral vector used for manufacturing of Zynteglo, the first approved gene therapy for ß-thalassemia.^43,96^ Hybrid LTR - hybrid long terminal repeat: the CMV enhancer and promoter replace the HIV U3 enhancer and promoter (allowing for Tat-independent translation), HIV R/U5 regions are preserved; ψ^+^ - extended packaging signal (carrying 2 stop codons to prevent readthrough from the CMV promoter); cPPT - central polypurine track (facilitates nuclear import of the viral preintegration complex); RRE – Rev responsive element (facilitates nuclear export of viral RNA in the packaging cell lines); I, II, III – human ß globin exons (the reverse orientation enables maintenance of the intron structure of the transgene; the Thr87Gln mutation confers anti-sickling properties without affecting hemoglobin function and enables tracking of therapeutic gene expression; a deletion in intron 2 is indicated by a triangle); HS2, HS3, HS4 – hypersensitive sites (HS) of the human ß-globin locus control region (stimulating ß-globin expression via looping to the ß-globin promoter); ppt – polypurine tract; ΔU3 – HIV LTR U3 region carrying a 400 bp deletion that results in self-inactivation after transduction (because the 3’ UTR serves as template for both LTRs); R – HIV LTR R region (including viral polyA signal); pA - rabbit ß-globin polyA signal (as additional safety measure).

A breakthrough for the clinical application of gene therapy has been the development of retroviral vectors. These vectors use the intrinsic capacity of retroviruses such as the Moloney Murine Leukemia Virus for efficient integration into the host genome and subsequent stable expression of viral genes. After the elimination of pathogenic sections of the viral genome, vectors derived from γ-retroviruses allow the transduction of hematopoietic stem cells and the expression of recombinant therapeutic genes in maturing and mature blood cells (Fig. 3). This revolutionary technique was first used for the treatment of inborn immunodeficiencies^46–48,51,52^ where already a low level expression of the therapeutic gene can correct the phenotype and where transduced cells acquire a selective advantage over cells that did not receive the therapeutic gene. In consequence, the first *ex vivo* gene therapy receiving marketing authorization in Europe in 2016 was that for ADA-SCID, a very rare severe combined immunodeficiency (SCID) caused by a deficiency of adenosine desaminase (ADA).^53^

In parallel, trials for the treatment of other rare immunodeficiencies were at least partially successful in terms of cure of the underlying disorder.^54–58^ Although gene therapy proved to be safe and effective in ADA-SCID, the therapeutic gene has been demonstrated to preferentially integrate in proximity of oncogenes in several other indications.^59–62^ The genotoxic potential of retroviruses was most drastically illustrated by the first series of patients treated for Wiskott-Aldrich syndrome: Seven of nine patients developed leukemia,^63^ a complication that scotched the enthusiasm for γ-retroviral vectors.

The insertional mutagenesis by γ-retroviral vectors is caused by the preferential integration of the vector into transcriptionally active regulatory regions of the host genome that results in a selection of modified cells with an activated oncogene that stimulates proliferation.^62–66^ The selectivity for transcriptionally active regions und thus the risk for an oncogenic insertion is mediated by the retroviral “long terminal repeats” (LTR).^67^ Because of the potential for the induction of leukemias, γ-retroviral vectors are no longer in clinical use.

## New self-inactivating (SIN) lentiviral vectors

Viral vectors that are used in current gene therapy approaches are derived from the human immunodeficiency virus (HIV) by deleting accessory virulence factors and regulatory genes from the viral genome. The envelope protein is exchanged by that of another virus (typically vesicular stomatitis virus- VSV) and expressed from a separate plasmid in the packaging cell line, as are the genes required for virus packaging, *gag*, *pol,* and *rev*. Deletions in the viral LTR preclude virus replication once the viral DNA is integrated in the host genome. The promoter function of the LTR is replaced by a constitutively active promoter that is independent from viral factors such as *tat*. Expression of the therapeutic gene is driven from the endogenous promoter and regulatory sequences.^68–73^ These *self-inactivating* (SIN) lentiviral vectors randomly integrate into the host genome without preference for regulatory regions.^74^ In addition, the loss of the LTRs limits their potential to activate genes in proximity of the integration site. With a vector copy number that typically reaches two to three per host genome, the risk of oncogene activation is statistically low, but cannot be completely excluded. In a growing number (>300) of patients treated for different genetic disorders in trials using lentiviral vectors, no malignancy related to lentiviral insertional mutagenesis has so far been detected.^43,48,74–82^ However, the number of patients treated for hemoglobinopathies with lentiviral gene therapy is still too low (approximately 100^43,44,83–85^) to be certain about the oncogenic risk in this specific indication.

## Challenges in gene therapy for ß-hemoglobinopathies

Although self-inactivating lentiviral vectors offer an efficient and hopefully safe way of manipulating hematopoietic stem cells, a clinically successful gene therapy still requires an optimization of all steps (Fig. 1).

Historically, the first source of stem cells for gene therapy of hemoglobinopathies was bone marrow. However, hematopoietic stem cells obtained from bone marrow are limited in quantity, typically requiring two or more harvesting procedures.^76,86^ In addition, chronic inflammation may impede the potential of proliferation and transduction of bone marrow stem cells in patients with SCD.^87^ For these reasons, hematopoietic stem cells collected from the peripheral blood have replaced bone marrow as a cell source for gene therapy.

For patients with thalassemia, the protocols for the mobilization of hematopoietic stem cells resemble that of other indications. However, the cell numbers required for the manufacturing process are much higher in comparison to that required for an autologous rescue after high dose chemotherapy and mobilization by chemotherapy is not indicated for a benign disorder. For this reason, the combination of G-CSF with (off-label) plerixafor is used most frequently.^43,44,88^ Stem cells mobilized by this combination are readily transduced by lentiviral vectors and produce more ß-globin per gene copy as compared to stem cells mobilized by other regimens. The higher expression level of the therapeutic gene after mobilization with the combination of G-CSF and plerixafor in comparison to other mobilization regimens may be related to a preferential integration into transcriptionally active chromatin regions.^89^

For patients with SCD, however, G-CSF is contraindicated, because it can precipitate fatal vaso-occlusive crises.^90,91^ Therefore, (off-label) plerixafor has been used as the only mobilization agent^92–94^ and results in stem cells that are well suited for transduction and expression of the therapeutic ß-globin.^89^

The apheresis procedure needs to account for the particular sedimentation properties of blood cells in patients with hemoglobinopathies. In comparison to other indications, the stem cell fraction typically needs to be collected with a much higher admixture of erythrocytes.^92^

In order to revert the transfusion requirement in TDT by gene addition, transduced stem cells must be able to differentiate into a sufficient number of erythroid precursors that produce as much ß-globin as a healthy ß-globin locus would do. The number of vector copies per genome in the peripheral blood after gene therapy is correlated with the number of vector copies in the manipulated stem cells.^43,44^ Although the level of hemoglobin expressed from the therapeutic gene increases with the proportion of transduced stem cells in the cell product and with the resulting vector copy number in the peripheral blood, the characteristics of the cell product do not fully predict the success of the gene therapy. One vector copy per periphal blood genome appears to be sufficient to render the patient independent from red blood cell transfusions.^43,44^

For this reason, the ideal cellular product with the lowest possible risk of insertional mutagenesis would be characterized by one vector copy in each cell. Although the success of gene therapy is determined by several factors and cannot easily be predicted, the quantity of therapeutic ß-globin that is required to correct TDT is reduced if the patient carries at least one ß^+^-globin allele that allows for a residual expression of ß-globin. In this context, it must be noted that the quantity of residual ß-globin chain synthesis depends on the type of the ß^+^-thalassemia mutation and varies from less than 5% to more than 20%.^89^ In order to reach the optimal vector copy number with a near to complete transduction of all cells in the graft, the transduction procedure itself needs to be optimized.^71,72^ Parameters that can improve transduction efficiency are the viral coat proteins,^95^ the use of growth factors and of polycations such as protamine,^96^ and the ratio of vector to target cells. Consequently, the enrichment of hematopoietic stem cells by the use of cell surface markers can greatly reduce the amount of lentiviral vector required.^97^ Before the graft can be re-transplanted, stringent quality controls have to ensure successful gene transfer into the host genome, viability and sterility.

In contrast to severe immunodeficiencies, the corrected cells in the hemoglobinopathies only slightly benefit from a proliferative advantage in comparison to cells with the uncorrected genetic defect.^45,98–100^ This is why the myeloablative conditioning of the recipient is of paramount importance. Unlike allogeneic stem cell transplantation, gene therapy does not require immunoablation with serotherapy or lymphotrophic chemotherapy such as fludarabine or cyclophosphamide. In clinical trials, either busulfan^42,43,76,101^ or the combination of treosulfan with thiotepa^44^ was used. An earlier trial with submyeloablative busulfan did not succeed but was hampered by a relatively low transduction efficiency resulting in copy numbers of approximately 0.5 per cell in the cellular product.^89,102,103^

The acute toxicity of gene therapy is determined by the myeloablative conditioning and comparable to that of high dose chemotherapy with mucositis, bacterial infections, bleeding and sinusoidal obstruction syndrome.^43^ In addition, chemoconditioning results in long term sequelae, most importantly infertility and treatment-induced malignancies. The latter has rarely been observed within the first ten years after conditioning with busulfan for allogeneic transplantation, but is a major concern in the long term follow-up.^104,105^ In order to avoid the toxicities of chemotherapy, alternative ways of myeloablation are being tested preclinically. These employ the depletion of CD45+ or CD117+ precursors with toxic immune conjugates^106–108^ and promise complete myeloablation with minimal systemic toxicity.

Most patients with SCD suffer from chronic vasculopathy with the risk of vasoocclusive crises at the time of gene therapy. As with allogeneic stem cell transplantation, prophylactic measures such as exchange transfusion and anticonvulsive prophylaxis are necessary to reduce the risk of acute complications.^109^

As for allogeneic stem cell transplantation, stem cells corrected by gene therapy are usually applied intravenously.^42,43,101^ A mouse model suggests that a direct intraosseous application may result in a more efficient homing of stem cells. Although some patients were successfully treated by the intraosseous application of genetically modified stem cells,^44^ neither of these application routes has been demonstrated to be superior over the other in a controlled trial.

After the infusion of genetically modified stem cells, several weeks of bone marrow aplasia carry the risk of infections and bleeding before hematopoiesis is finally restored. The full expression of the therapeutic gene can be expected after 3 to 6 months and can be correlated with a reduction or, ideally, complete resolution of the transfusion requirement.^43,44^ A possible reason for the delay between application of the graft and full expression of therapeutic hemoglobin may be a selection for clones that most efficiently express the transgene. This clonal evolution of hematopoiesis derived from manipulated stem cells can be monitored by integration site analysis that demonstrates dynamic changes in the clonal composition even after years.^43^ Vectors that code for a ß-globin with an amino acid substitution allow for the quantification of the expression of the therapeutic gene in relation to endogenous ß-globin by hemoglobin separation.^71,96^ In addition, the physicochemical properties of the therapeutic ß-globin can be modified by amino acid substitutions to interfere with the polymerization of HbS. Similarly to an increased expression of HbF, the expression of such therapeutic ß-globins tackles the pathophysiology of SCD at the very root.^110,111^

## Clinical results of “gene addition” for transfusion-dependent thalassemia and for sickle cell disease

A first patient treated with lentiviral gene addition for TDT in 2007 reached independence from transfusions, but showed clonal hematopoiesis likely caused by insertional mutagenesis which persisted for years.^43,112^ Subsequently, with vectors that were modified to reduce the risk of insertional mutagenesis, several trials demonstrated a success of gene therapy for thalassemia without inducing clonal hematopoiesis. Both made use of a lentiviral gene addition therapy, either with the vector BB305^42,43,113^ or the GLOBE vector.^44^ Most important predictors for the success of gene therapy were genotype and patient age.

The HGB-204 and HGB-205 trials run by bluebird bio reported that 12 of 13 evaluable patients with a non-ß^0^/ß^0^ genotype became independent from transfusions (median duration 38 months for HGB-204).^43,83^ The subsequent trial HGB-207 used an improved transduction procedure and reached independence from transfusions for at least 6 months in 10 of 11 patients.^114^ Based on these results, the European Medicines Agency conditionally licensed gene therapy by lentiviral gene addition (Zynteglo™) for patients with TDT aged 12 years or older and a non-ß^0^/ß^0^ genotype for whom no HLA-matched sibling is available as a stem cell donor.^115^

Treatment of TDT in patients with a ß^0^/ß^0^ genotype proved to be more difficult: In the HGB204 trial, transfusion frequency was reduced in all patients, but only four of eight evaluable patients reached complete transfusion independence.^43,83^ This suboptimal albeit promising result was attributed to an insufficient transduction efficiency with low vector copy numbers in the hematopoietic stem cells and to an insufficient proportion of transduced stem cells. While the follow-up is still too short to draw final conclusions, first results of the succeeding trial HGB-212 show that with an improved transduction procedure three of four TDT patients with a ß^0^/ß^0^ genotype were off transfusions for at least six months after gene therapy.^116,117^ It must be noted, however, that even patients who become transfusion independent following gene therapy may not have been completely cured. In a study of 8 such patients there was remaining, albeit improved ineffective erythropoiesis and a marker profile indicating disturbed iron homeostasis likely resulting in inappropriate iron resorption.^118^ Therefore, these patients may have to be monitored for extramedullary erythropoiesis and may still require (costly) iron chelation.

With the GLOBE vector, four of 6 children but none of 3 adults with TDT became independent from transfusions. However, only one of these patients had a non-ß^0^/ß^0^ genotype with relevant residual synthesis of endogenous ß-globin and was successfully treated. The authors attributed the difference between children and adults to a defect of the stem cell niche induced by long-term transfusions.^44,119^

In SCD, successful gene therapy must reduce the frequency and severity of vaso-occlusive complications such as pain crises or acute chest syndrome and ultimately prevent or even reverse end organ damage. Patients with compound heterozygosity for the HbS mutation and a second allele resulting in the persistence of γ-globin until adulthood are free of symptoms if all erythrocytes contain at least 30% of HbF.^16^ In analogy, a pancellular expression of a therapeutic ß-globin chain interfering with the polymerization of HbS to the same level is expected to blunt the symptoms and freeze progression of SCD.

This goal was partially reached in the first patient treated by gene addition with the lentiviral vector BB305.^42,113^ In further patients, however, the vector copy number and subsequently the expression of anti-sickling ß-globin did not suffice to clearly improve the complications of SCD.^76,113^ As for the TDT trials, the gene therapy protocol for SCD needed optimization at several levels. With the use of plerixafor for mobilization and improved technology for transduction, the most recently treated group of patients expressed approximately 6 g/dl of therapeutic hemoglobin, distributed in a pancellular fashion. As expected, with this expression level of the anti-sickling ß-globin, laboratory parameters for hemolysis normalized and the frequency of vasoocclusive complications was reduced.^76,85,101^

## Gene therapy beyond gene addition

**Figure 4:**
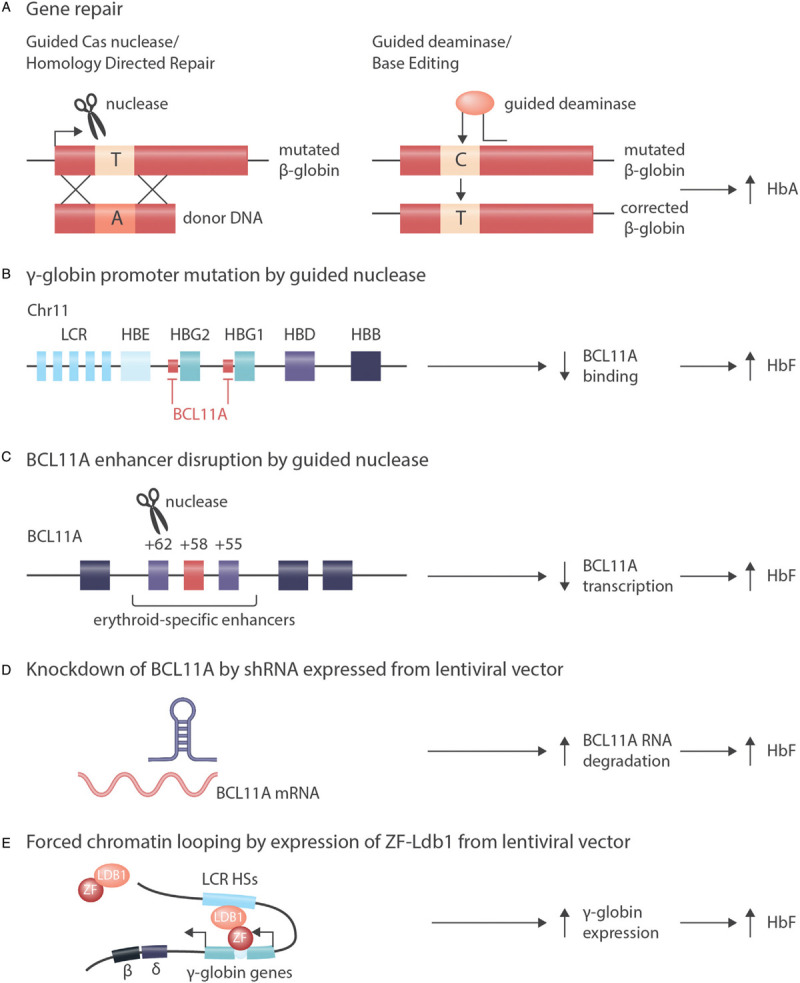
**Alternatives to gene addition for correction of hemoglobinopathies.** A: In cells with active HDR (homology directed repair) double strand breaks introduced by nucleases can be repaired with the help of a template, resulting in the correction of pathogenic mutations.^41^ Because a specific template is needed for each mutation and because HDR is not active in hematopoietic stem cells, this approach is not in clinical use for the hemoglobinopathies. An alternative technique of direct correction of pathogenic mutations is “base editing” by deaminases that result in C to T or G to A changes in a sequence context defined by a guide RNA.^122^ While this technique does not require any endogenous DNA repair activity, it suffers from limited specificity and thus from off target effects. B: Induction of γ-globin expression by introduction of mutations that activate the expression of γ-globin as can be naturally observed in HPFH (hereditary persistence of fetal hemoglobin), presumably by abrogating BCL11A binding.^130,131^ C: Induction of γ-globin expression by disruption of an erythroid-specific BCL11A enhancer.^132,136–138^ Approaches B and C can be used for all ß-hemoglobinopathies and rely on non-homologous end joining or microhomology-mediated end joining that are active in hematopoietic stem cells. D: Induction of γ-globin expression by depletion of BCL11A via shRNA expressed from a lentiviral vector.^93,133^ E: Induction of γ-globin expression by forced chromatin looping mediated by a Zink-Finger/Ldb1-fusion protein expressed from a lentiviral vector.^146^ Approaches D and E can be used in principle for all ß-hemoglobinopathies and do not rely on endogenous DNA repair activity.

Besides “gene addition,” several other strategies either aim at a direct repair of the pathogenic mutation or at neutralizing the pathogenic mutation by the introduction of genomic changes in *trans* (Fig. 4). While preclinical studies are far advanced, only very limited clinical experience with these techniques is available (Table 1).

**Table 1:**
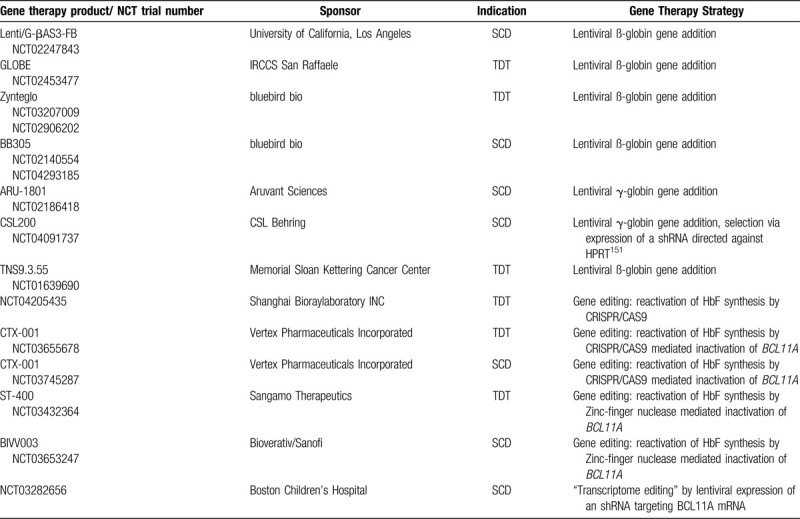
Active Clinical Trials for Gene Therapy of ß-hemoglobinopathies (https://www.clinicaltrials.gov, Accessed 09.04.2020, Filtering Criteria Were “Thalassemia OR Sickle Cell Disease” AND “Gene Therapy” AND “Recruiting OR Active/Non-recruiting”).

A direct correction of pathogenic mutations (Fig. 4A) could be achieved by inserting a therapeutic DNA fragment with the help of *homology-directed repair* (HDR). However, the poor efficiency of HDR in hematopoietic stem cells precluded its use for gene therapy of hemoglobinopathies so far.^120^ Specific base editing can alternatively be achieved by expressing cytidine deaminases that are guided by small RNAs to a specific target sequence and result in C>T or G>A mutations.^121,122^ Due to restrictions in the targeting of the “base editors” to a target sequence and because of off-target effects, this technique has not been employed clinically so far.

While for SCD the direct repair of the underlying mutations appears attractive once the technical issues are solved, in TDT the direct repair of the underlying ß-globin mutations is cumbersome because more than 300 different mutations in the ß-globin gene resulting in a thalassemia phenotype would need to be addressed.

This is why all genome editing approaches that are currently being developed do not target the ß-globin gene but aim at the re-activation of the γ-globin gene and at the expression of HbF. Despite the difference in oxygen affinity when compared to HbA, HbF is a fully functional globin chain even in adult life and can replace the lacking HbA in ß-thalassemia.^15^ HbF can prevent or limit the polymerization of deoxygenized HbS in SCD, offering a therapeutic target for both ß-hemoglobinopathies. Patients with hereditary persistence of fetal hemoglobin (HPFH) demonstrate that HbF can substitute for HbA without major functional limitations^15^ and protect from polymerization of HbS if co-expressed with the HbS mutation.^16^ Such an indirect strategy of modifying globin synthesis thus carries the potential of curing both TDT and SCD,^123,124^ although cure of ß-thalassemia by this strategy will require a more pronounced reactivation of γ-globin chain synthesis than will likely be required for SCD.

The techniques used for genome editing in hematopoietic stem cells do not aim at reverting or introducing a specific mutation, but rather at the inactivation of certain DNA sequences. Such an inactivation can be achieved in a sequence-specific manner without the need for endogenous homology-directed repair activity by the CRISPR/Cas9 system. The CRISPR/Cas9 system is making use of prokaryotic enzymes that recognize and degrade DNA sequences introduced into bacteria by bacteriophages.^125,126^ Specific guide RNAs recruit the endonuclease Cas9 to specific sites of the genome, where Cas9 introduces double strand breaks that will be repaired by nonhomologous end joining (NHEJ). Imprecisions in the repair process result in small insertions or deletions that inactivate the target gene.^127,128^ An alternative technique of introducing targeted double strand breaks into DNA involves nucleases that directly bind to the target sequence without the need of a specific guide RNA. Double strand breaks introduced by such **T**ranscription **A**ctivator-**L**ike **E**ffector **N**ucleases (TALEN) or by Zink finger enzymes inactivate the respective gene as do double strand breaks introduced by Cas9.^129^

Several strategies employ genome editing to boost HbF expression, most directly by the introduction of activating mutations in the γ-globin promoter that abrogate binding of postnatal repressors (Fig. 4B).^130,131^ The most frequently modified target of genome editing for the ß-hemoglobinopathies is *BCL11A*. *BCL11A* encodes a transcription factor that is required to turn off γ-globin expression during the perinatal hemoglobin switch. Inactivation of *BCL11A* in erythroid precursors results in an increased synthesis of HbF.^132–134^ Similarly, disruption of the interaction of LRF, another transcription factor promoting the hemoglobin switch, with the γ-globin promoter induces HbF expression.^135^

*BCL11A* can be specifically inactivated (Fig. 4C) in erythroid cells by targeting a TALEN nuclease^136,137^ or a CRISPR/Cas9 nuclease^132^ against a lineage specific enhancer of *BCL11A*. This lineage specificity^138^ is important, because BCL11A is not only crucial for erythroid, but also for lymphoid^139^ and neuronal^140^ differentiation. If *BCL11A* is disabled in erythroid precursors derived from edited hematopoietic stem cells, there is a robust induction of HbF.^132,136,137^ Very first patients with TDT and with SCD were treated with a CRISP/Cas9-mediated inactivation of *BCL11A* (Table 1, NCT03745287 and NCT03655678). While follow up is short and numbers small, preliminary results are encouraging.^141^ Similarly, disruption of *BCL11A* in hematopoetic stem cells by Zink finger nucleases resulted in HbF induction both in erythroid cells and in first patients with TDT who received these stem cells.^142,143^

Another way of inducing HbF by repressing *BCL11A* makes use of the expression of short hairpin (sh)RNAs that are introduced into hematopoietic stem cells by a lentiviral vector (Fig. 4D). By specific hybridization to and degradation of the target mRNA, these shRNAs downmodulate the expression of their target gene. Inducing HbF by downmodulating *BCL11A* in hematopoietic stem cells by shRNA is currently explored as treatment of SCD.^133^ Preliminary results of this “transcriptome editing” approach in three patients with SCD who were followed at least 6 months after gene therapy showed a lineage-specific induction of HbF and an improvement of hemolysis.^93^

Yet another alternative to “genome editing” is the expression of an engineered fusion protein between Ldb1 and a Zink finger protein from a lentiviral vector (Fig. 4E) that forces the ß-globin locus control region into proximity of the γ-globin gene. As a result, HbF expression is reactivated and exceeds that of HbS *in vitro*^144–146^—a situation compatible with cure of SCD if achieved *in vivo*.

A combination of more than one of the mentioned strategies would have the potential to optimize the expression of a therapeutic gene product. Such a combination may for instance include the expression of ß-globin via lentiviral gene addition and at the same time repressing *BCL11A* via shRNAs encoded by the same vector and could result in a success rate that robustly outperforms that of allogeneic stem cell transplantation.^147^

## Perspectives

The current standard of care for TDT and SCD is a disease modifying treatment with red blood cell transfusions, iron chelation and pharmacologic induction of HbF that needs to be combined with the treatment of symptoms and complications. Recently, additional treatments for TDT that aim at improving the survival of erythroid precursors have emerged.^148^ However, the only established curative option is allogeneic stem cell transplantation that, depending on patient age and the type of donor, is associated with relevant morbidity including graft versus host disease, infertility and other long term complications.^104,105^ While transplantation from an HLA-matched sibling is widely accepted as standard of care, for most patients such a donor is not available and transplantation from alternative donors still carries a risk of treatment-related mortality of 10% or higher that is considered not acceptable for non-malignant disorders.^34,37,109,149^

Gene therapy bypasses two of the most relevant shortcomings of allogeneic transplantation—limited donor availability and risk of GvHD—while possibly offering cure in 80% of patients with TDT with a non-ß^0^/ß^0^ genotype. So far, no treatment-related mortalities have been reported after gene therapy for hemoglobinopathies. These results have led to the licensing of the first gene therapy for transfusion dependent thalassemia in Europe and offer the chance to assess the potential of gene therapy under real world conditions and in a larger number of patients.

Two major obstacles currently preclude the widespread use of gene therapy. First, current protocols still require myeloablative conditioning. Even if in comparison to allogeneic stem cell transplantation the cumulative dose of chemotherapy is reduced, long term sequelae such as infertility and the risk of secondary neoplasms will likely remain a challenge. Second, the sophisticated logistics and the costs of gene therapy^150^ limit its use in patients and health care systems with sufficient resources while prohibiting its use in countries with a high prevalence of hemoglobinopathies which cannot provide the required infrastructure and resources. Obviously, the development of conditioning regimens with reduced toxicity and lowering the costs of gene therapy are the most important steps required to bring this treatment options to more than a small number of selected patients.
